# Fourteen-Year-Old Boy With Intracranial Internal Carotid Artery Aneurysm Presenting as Mood Disorder

**DOI:** 10.7759/cureus.18324

**Published:** 2021-09-27

**Authors:** Kanakka Hewage Dammika Madhusankha, Dilruk Rathnayaka, Malinga Samaranayake, Mahima Dharmasiri, Ranjith Wickramasingha

**Affiliations:** 1 Department of Emergency Medicine, National Hospital Kandy, Kandy, LKA; 2 Department of Emergency Medicine, National Hospital Sri Lanka, Colombo, LKA; 3 Department of Neurosurgery, National Hospital Kandy, Kandy, LKA

**Keywords:** pediatric intracranial aneurysms, sertraline, sah, internal carotid artery aneurysms, mood disorder, structural brain lesion, aneurysmal subarachnoid hemorrhage

## Abstract

Intracranial aneurysms (IA) are very uncommon to find in the pediatric population. If present, it is usually associated with other genetic illnesses. Most of the intracranial aneurysm has been presented due to mass effect of the aneurysm or as subarachnoid hemorrhage. We report this young Asian kid who has had a possible ictus of subarachnoid hemorrhage (SAH) with depressive symptoms, later presenting with classic features of SAH due to rupture of intracranial internal carotid artery aneurysm. The use of Sertraline to treat depressive episodes may aggregate the condition due to its antiplatelet effect. The patient showed significant improvement following microsurgical clipping of the aneurysm. This case is another example that young patients coming with the first episode of mood disorder should be carefully excluded for other intracranial pathology, including intracranial aneurysms, before coming to the final diagnosis.

## Introduction

Intracranial aneurysms (IA) are rare in the pediatric population. Their clinical presentation and associations are different from the adult population. As stated by the Cooperative study on intracranial aneurysms and subarachnoid hemorrhage, which studied more than 6000 cases, only four patients were less than 20 years old [1]. The commonest sites of intracranial aneurysms in the pediatric population are intracranial internal carotid artery and the commonest associations are polycystic kidney disease, coarctation of the aorta, fibromuscular dysplasia, Marfan syndrome, and Ehlers-Danlos syndrome [2,3]. Most of the patients presented with subarachnoid hemorrhage and other presentations are due to the mass effect. We write this young kid who has had a possible ictus of SAH with depressive symptoms later presenting with classic features of SAH due to rupture of intracranial internal carotid artery aneurysm.

## Case presentation

A 14-year-old Asian male presented to the psychiatric clinic with a one-month history of on and off left-sided headaches, photophobia, irritability, feeling sadness, and poor sleep. History did not reveal past history of other somatic or psychiatric illness and he was not taking any medication. He was not drug-addicted.

His physical examination was normal. Routine blood tests did not find abnormalities. His psychiatric assessment revealed that he was alert, well oriented with depressive signs. The patient denied suicidal thoughts. The neurological examination did not reveal focal neurological signs or cranial nerve deficits. According to the neuropsychological assessment, he had mild cognitive impairment, moderate to severe depressive episode, and mild anxiety. He was diagnosed to have an adolescent depressive episode, which was treated with Sertraline and Flunarizine. Two weeks later he presented to the emergency department with sudden onset of severe occipital headache and vomiting. There were no focal neurological deficits and Glasgow Coma Score was 15.

**Figure 1:**
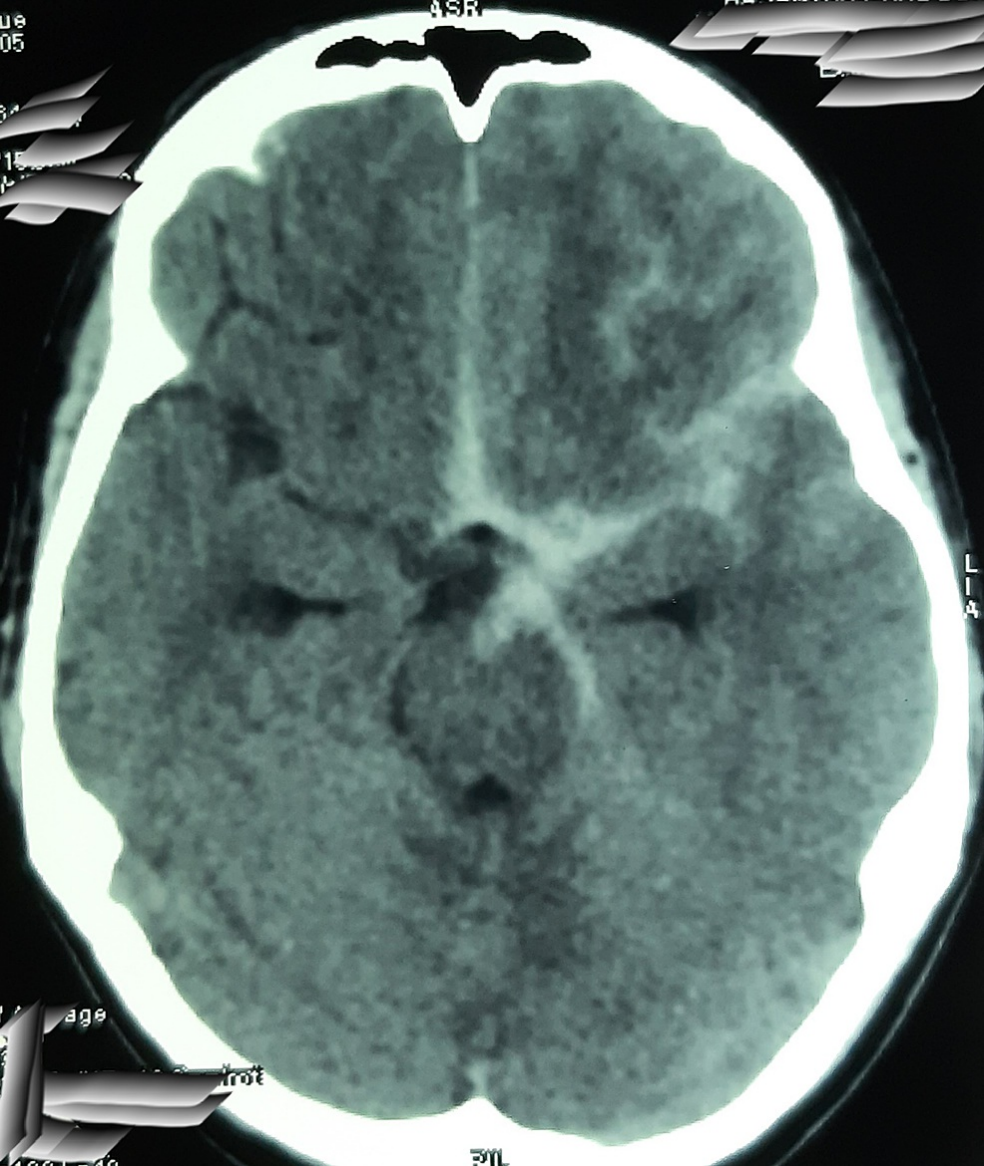
Non-contrast CT brain axial cuts showing subarachnoid hemorrhage

**Figure 2:**
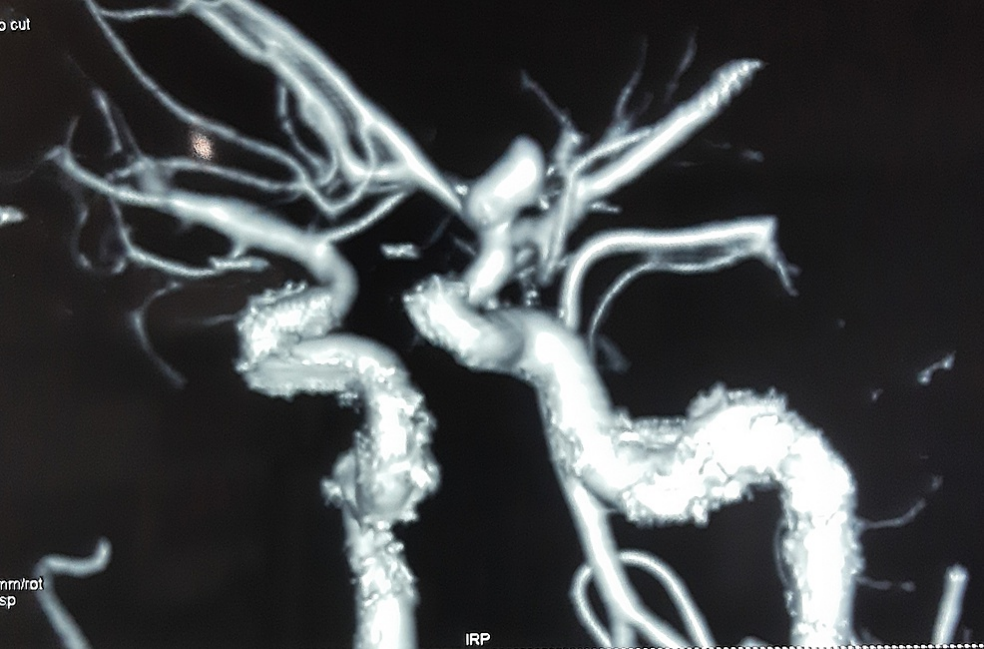
Digital subtraction angiography demonstrating left internal carotid artery aneurysm

**Figure 3:**
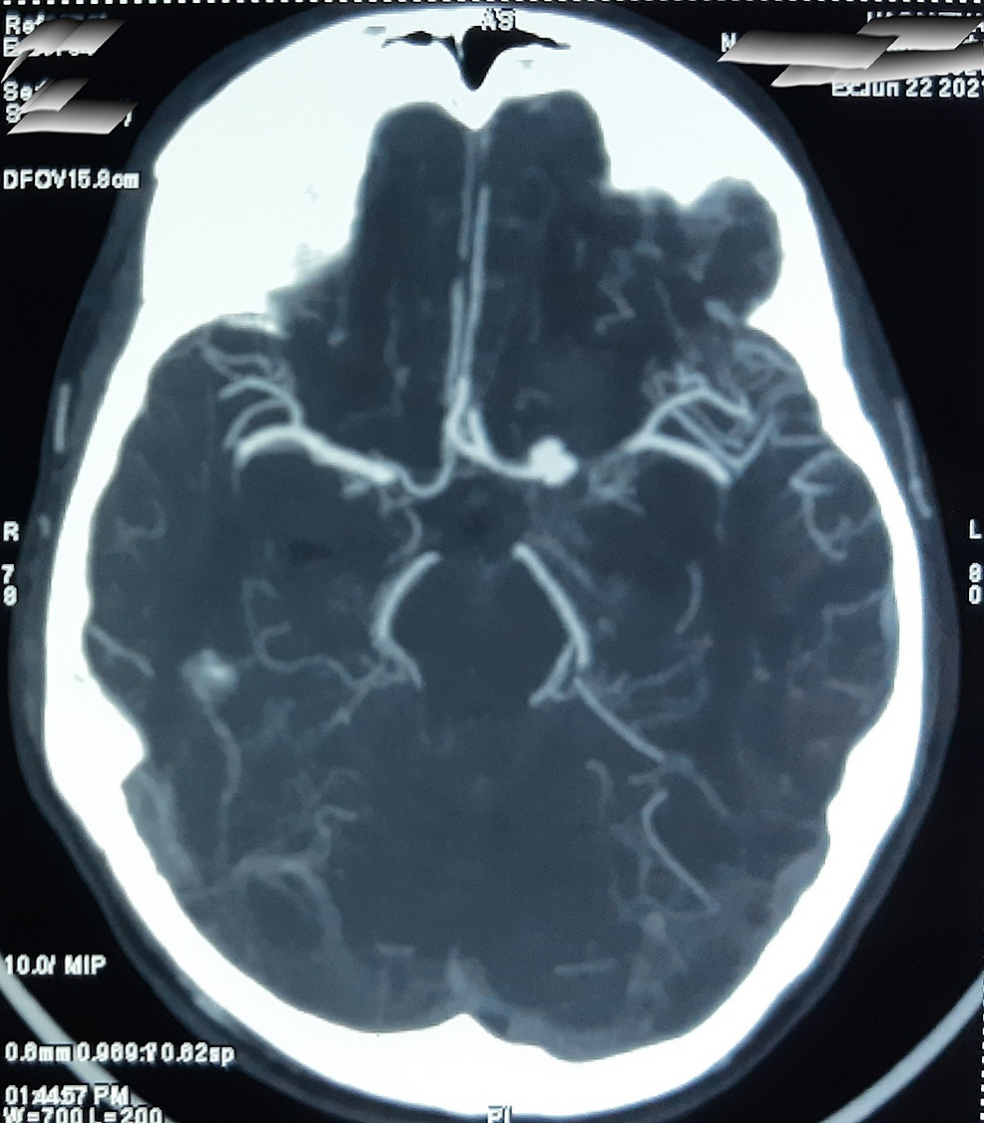
Contrast CT angiogram demonstrating left internal carotid artery aneurysm

The non-contrast computed tomography (CT) of the brain was done to rule out acute brain insult. The CT brain demonstrated thick subarachnoid hemorrhage (Figure 1). Subsequent contrast CT angiogram and digital subtraction angiogram (DSA) confirmed the diagnosis of a 5.5 × 9.2 mm^2^ saccular aneurysm originating from the bifurcation of the left internal carotid artery (Figures 2-3). The neurosurgical team did the microsurgical clipping of the aneurysm. The immediate postoperative period was uncomplicated and the patient was discharged from the hospital seven days after the surgery. On psychiatric evaluation two weeks after the surgery, the patient showed improvement in sleep, less anxiety, and depressive symptoms. As the depressive symptoms were resolving, he had stopped taking Sertraline. On neurological assessment, he was alert and well oriented, without any neurological deficits. During the psychiatric assessment one month after the surgery, his depressive symptoms were resolved and he enjoyed his day-to-day activities. On psychological assessments, his cognitive functions were within normal limits, and we did not detect depressive symptoms or neurological deficits.

## Discussion

This is a case of an uncommon presentation of a 14-year-old boy, who was recently diagnosed to have adolescent depression and migraine, presenting to the emergency department with subarachnoid hemorrhage following spontaneous rupture of intracranial aneurysm. It is uncommon to find ruptured cerebral aneurysms in otherwise healthy early adolescents. Also, the exact reason for the association between depressive episode and internal carotid artery aneurysm at the bifurcation cannot be discerned. Because there is a close relationship of bifurcation of the internal carotid artery to the frontal lobe and its blood supply to the frontal lobe. It is well reported in the literature that structural and vascular anomalies within the frontal lobe and limbic system can cause depressive disorders [4]. However, our patient did not have any compressive features, and there were no obvious ischemic changes noted in the frontal lobe due to thrombosis and microemboli from the aneurysm. Our patient was taking Sertraline and Flunarizine for his recently diagnosed depressive episode. Sertraline is a selective serotonin receptor inhibitor (SSRI), which can cause hypertension and antiplatelet effects [5]. Hypertension and antiplatelet drugs facilitate subarachnoid hemorrhage.

Undiagnosed patients with un-ruptured intracerebral aneurysms can present with a neurological or neuropsychiatric illness like a migraine without aura, focal and generalized seizures, frontal lobe syndrome, and visual impairment [6]. Intracranial internal carotid artery aneurysm may present with diplopia, retro-orbital pain, and unilateral headache mimicking migraine without aura. It has been well reported that other intracranial pathologies such as brain tumors and arteriovenous malformations may present as neuropsychiatric illnesses. Treating the underlying aneurysm can alleviate these symptoms and improve a patient's clinical outcome.

## Conclusions

This case reveals an uncommon association between newly diagnosed depressive episode and intracranial internal carotid artery aneurysm in a 14 years old otherwise healthy boy. Also, this case shows that young patients coming with the first episode of mood disorder should be carefully evaluated for underlying intracranial pathology as well as common associations of intracranial aneurysms.
